# Integrating gated recurrent unit in graph neural network to improve infectious disease prediction: an attempt

**DOI:** 10.3389/fpubh.2024.1397260

**Published:** 2024-05-20

**Authors:** Xu-dong Liu, Bo-han Hou, Zhong-jun Xie, Ning Feng, Xiao-ping Dong

**Affiliations:** ^1^Faculty of Information Technology, Beijing University of Technology, Chaoyang District, Beijing, China; ^2^Key Laboratory of Computational Intelligence and Intelligent Systems, Beijing University of Technology, Chaoyang District, Beijing, China; ^3^Office of International Cooperation, Chinese Center for Disease Control and Prevention, Chaoyang District, Beijing, China; ^4^National Institute for Viral Disease Control and Prevention, Chinese Center for Disease Control and Prevention, Chaoyang District, Beijing, China; ^5^National Key-Laboratory of Intelligent Tracking and Forecasting for Infectious Disease, National Institute for Viral Disease Control and Prevention, Chinese Center for Disease Control and Prevention, Chang-Bai, Beijing, China

**Keywords:** artificial intelligence technology, graph neural network, gated recurrent unit, infectious disease, time series prediction

## Abstract

**Objective:**

This study focuses on enhancing the precision of epidemic time series data prediction by integrating Gated Recurrent Unit (GRU) into a Graph Neural Network (GNN), forming the GRGNN. The accuracy of the GNN (Graph Neural Network) network with introduced GRU (Gated Recurrent Units) is validated by comparing it with seven commonly used prediction methods.

**Method:**

The GRGNN methodology involves multivariate time series prediction using a GNN (Graph Neural Network) network improved by the integration of GRU (Gated Recurrent Units). Additionally, Graphical Fourier Transform (GFT) and Discrete Fourier Transform (DFT) are introduced. GFT captures inter-sequence correlations in the spectral domain, while DFT transforms data from the time domain to the frequency domain, revealing temporal node correlations. Following GFT and DFT, outbreak data are predicted through one-dimensional convolution and gated linear regression in the frequency domain, graph convolution in the spectral domain, and GRU (Gated Recurrent Units) in the time domain. The inverse transformation of GFT and DFT is employed, and final predictions are obtained after passing through a fully connected layer. Evaluation is conducted on three datasets: the COVID-19 datasets of 38 African countries and 42 European countries from worldometers, and the chickenpox dataset of 20 Hungarian regions from Kaggle. Metrics include Average Root Mean Square Error (ARMSE) and Average Mean Absolute Error (AMAE).

**Result:**

For African COVID-19 dataset and Hungarian Chickenpox dataset, GRGNN consistently outperforms other methods in ARMSE and AMAE across various prediction step lengths. Optimal results are achieved even at extended prediction steps, highlighting the model’s robustness.

**Conclusion:**

GRGNN proves effective in predicting epidemic time series data with high accuracy, demonstrating its potential in epidemic surveillance and early warning applications. However, further discussions and studies are warranted to refine its application and judgment methods, emphasizing the ongoing need for exploration and research in this domain.

## 1 Introduction

Multivariate time series forecasting plays a crucial role in various real-world scenarios such as transportation forecasting (1, 2), supply chain management (3), energy allocation (4, 5) and financial investment (6). The time series prediction is involves forecasting future values based on historical data points in a sequential order. This makes the statical method and supervised learning method, comparing with reinforcement learning (7, 8) or unsupervised learning methods, are more suitable for this task. In the field of public health, the problem of acute epidemic forecasting is of great relevance as a typical multivariate time series forecasting: if the future evolution of acute epidemic data can be estimated quickly and accurately for each geographic region, the forecasting results can be used as a reference to help governmental agencies make decisions on policy formulation and material deployment, and thus prevent the development and spread of epidemics.

The field of epidemiology and public health research has witnessed a large number of studies on time series prediction of infectious diseases which revealed the requirement of prediction method in the field of epidemiology and public health research. A selection of notable works has contributed to this progress, showcasing innovative approaches and methodologies for forecasting and managing disease outbreaks. For instance, Pinto et al. (9) applied a regressive model to estimate intervention effects over time by comparing rates of congenital syphilis. Cori et al. (10) presents a novel tool for tracking the spread of diseases by estimating time-varying reproduction numbers. Du et al. (11) focus on the research of serial interval of COVID-19 which contribute to the foundation of transmission dynamics of COVID-19 and is essential for effective prediction and control measures. However, when facing the outbreak of acute epidemic, the traditional transmission dynamics may be uncapable to prediction task. For example, in 2020, Ioannidis et al. (12) found that traditional transmission models failed in forecasting of COVID-19. And many research attempt to apply machine learning method to handle the problem. Dairi et al. (13) compared 7 kinds of neural network in the prediction of the number of COVID-19 cases. In fact, the neural networks were also applied to the prediction problem of other epidemics. Sanchez-Gendriz et al. (14) applied Long Short-Term Memory (LSTM) network in the prediction of dengue outbreak in Natal, demonstrates the potential of neural network in disease surveillance at a local scale. And It is worthwhile to research the potential of neural network in epidemic time series data prediction.

Early time series forecasting research mainly relied on traditional statistical models, including historical average (HA), autoregressive (AR), autoregressive integrated moving average (ARIMA) (15), VAR (16), and fuzzy methods (17). All of these statistical models rely on inherent *a priori* assumptions and require an artificial analysis of the characteristics of the study population to determine the applicability of the forecasting method.

Accurate prediction of multivariate time series data is a challenging type of time series forecasting problem, because both the correlation between the time nodes within each single time series and the correlation between the time series need to be considered comprehensively. During the outbreak of an infectious disease in a certain area, the changes in the number of active cases, on one hand, is related to the number of existing active cases in the locality or previous epidemic data. For instance, the outbreak of some infectious diseases has obvious seasonality, and by referring to the changes in active cases in previous years, one can roughly predict the current trend of active case changes. The data from a certain point or period in the time series is related to the data from the current or future time points, which reflects the correlation between the time nodes within each single time series. On the other hand, the number of active cases in a certain area may be related to the case numbers in neighboring areas or areas with frequent personnel movement. These time series may exhibit leading, lagging, or even synchronous trends, which demonstrates the correlation between different points within the time series. Deep learning models provide new ideas for this problem: on the one hand, Temporal Convolutional Network (TCN) (18) has excellent results in single time series prediction. Recurrent Neural Network (RNN) based methods (19–21) such as LSTM (Long Short-Term Memory) (22), Gated Recurrent Unit (23), Gated Linear Unit (GLU) (24) have good results in single time series prediction. GLU can effectively capture and learn the correlation and nonlinear features among time nodes within a time series (24). Han et al. (25) compared the prediction effects of ARIMA, deep neural network (DNN), and LSTM (Long Short-Term Memory) network for occupational pneumoconiosis data in Tianjin, China, and proved that LSTM (Long Short-Term Memory) can effectively predict occupational pneumoconiosis data, and at the same time has an advantage in prediction accuracy comparing to DNN and ARIMA. There is an advantage in prediction accuracy. However, most of these models ignore the dependencies between multiple variables and can only capture and learn the features within a single time series in isolation, which makes them perform poorly in practical multivariate time series prediction problems.

Meanwhile, in the problem of mining relationships between sequences, Yu et al. used matrix decomposition to model the relationship between multiple time series (26). Discrete Fourier Transform (DFT) is also useful in time series analysis by introducing it. For example, State Frequency Memory Network (27) combines the advantages of DFT and LSTM (Long Short-Term Memory) for stock price prediction; Spectral Residual model (28) utilized DFT to achieve desirable results in time series anomaly detection. Another important aspect of multivariate time series forecasting is modeling the correlation between multiple time series. For example, in traffic prediction tasks, neighboring roads naturally interact with each other. The state-of-the-art models rely heavily on graph convolution networks (GCNs) derived from graph Fourier transform (GFT) theory (29). These models (1, 2) directly stack GCNs and temporal modules (e.g., LSTM (Long Short-Term Memory), GRU (Gated Recurrent Unit)), which require predefined graph-structured relationships between sequences. By simultaneously capturing the dependencies between time nodes within each single sequence and between different time series to improve the learning of features of the time series and thus improve the prediction accuracy. Convolutional Neural Network (CNN) has a good performance in learning local features (30). There have been several methods to model spatial features using CNNs (31–35). Ma et al. (34) used deep CNN for traffic speed prediction. Huang et al. (36) tried to use transformer to predict multiple time series variables and obtained good prediction results.

The introduction of GRU (Gated Recurrent Unit) units provides better learning and fitting capabilities in the time domain compared to the linear units used in general GNN (Graph Neural Network) research methods. In addition, the above processes are modularized when implemented. Individual modules can be connected in series by shortcut connection to further improve the prediction accuracy of the neural network by constructing a deep network. Due to the advantages of RNN methods, such as LSTM (Long Short-Term Memory) and GRU (Gated Recurrent Unit), comparing with normal feed-forward neural networks, exist clear advantages in time series prediction, there have been a large number of attempts to use RNNs combined with GNNs (Graph Neural Networks), CNNs, or other neural network architectures to predict multivariate time series: Lv et al. (33) combined RNN with CNN, where the RNN are responsible for mining and learning intra-sequence time series within single sequence features, and CNN captures the relationships between sequences. Luo et al. (37) introduced GRU (Gated Recurrent Unit) into GCN to predict the change of gas composition in transformer oil during transformer operation. Zhang et al. (38) proposed ST-ResNet based on residual convolutional network for crowd flow prediction. Shi et al. (20) combines convolutional network with LSTM (Long Short-Term Memory) network to extract spatio-temporal information separately.

Graph neural networks have also yielded many results in capturing dependencies among unstructured data (1, 2, 7, 29, 39–43). DCRNN (1) and STGCN (2) are two of the first studies to introduce graph convolutional networks into spatio-temporal data prediction for better modeling of spatial dependencies. ASTGCN (40) adds an additional attention layer to capture the dynamic change of spatio-temporal dependencies. Adaptive learning of adjacency matrices can also be introduced to solve problems that require predefined graphs for adjacency matrices (35, 39, 41, 42).

However, the previous studies have never processed the time series data from three domains and they have hardly ever been applied in dealing with epidemic time series data predicting problems. But they provide the fundamental framework of the GNN (Graph Neural Network) and GRU (Gated Recurrent Unit) methods and prove the effectiveness of the methods so that we can reform the methods to cater the requirement that introducing GRU (Gated Recurrent Unit) units into GNN (Graph Neural Network) to achieve better results in time series data prediction problems.

The goal of this study is to try to introduce a GRU (Gated Recurrent Unit) layer in the graph neural network to enable the network to better capture and learn the relationship of each single time node within a sequence and the correlation between individual time series. Specifically, after this change, the neural network is able to learn features and make predictions from multivariate time series data in the frequency, spectral, and time domains: after GFT and DFT, it is easier to perform convolution and graphical convolution operations on the time series in the frequency and spectral domains respectively, which in turn allows for more effective predictions. The introduction of GRU (Gated Recurrent Unit) units provides better learning in the time domain compared to linear units used in the general GNN (Graph Neural Network) research methods.

## 2 Methods

**Figure 1:**
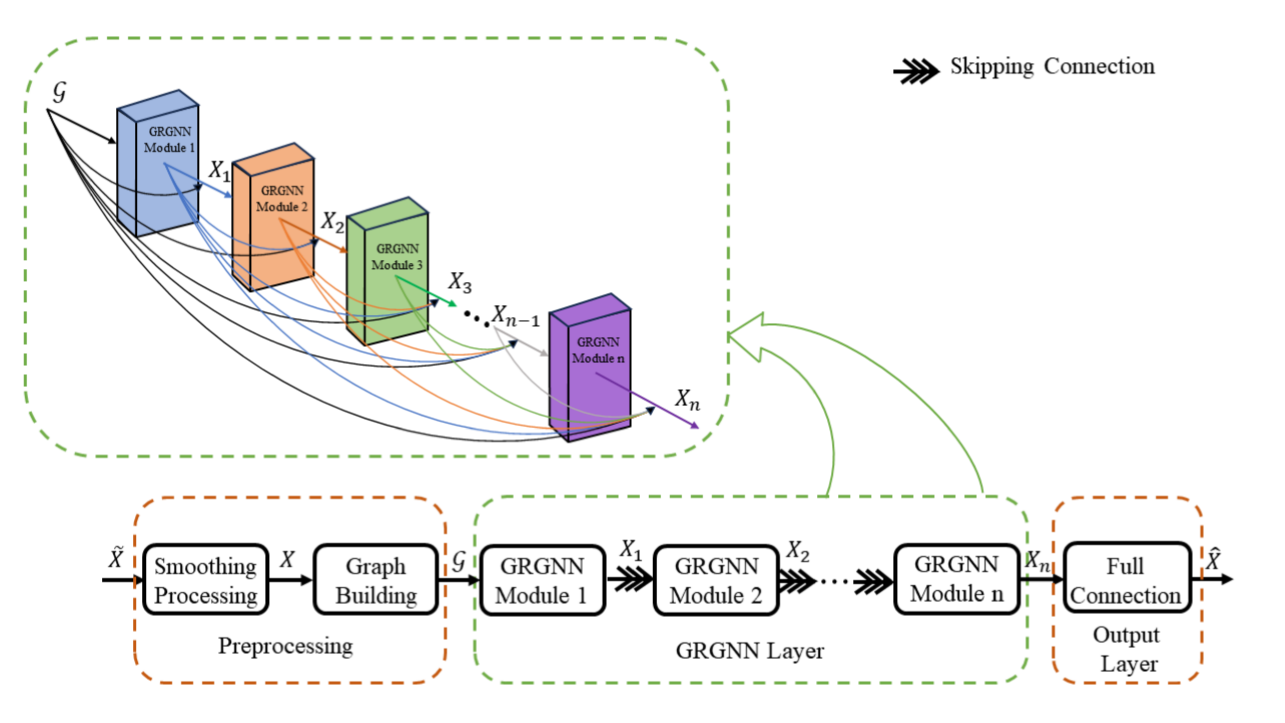
The overall structure of the improved GRGNN network.

The overall structure of the improved GNN (Graph Neural Network) network (later referred to as GRGNN) with the introduction of GRU (Gated Recurrent Unit) consists of three parts: the preprocessing layer, the GRGNN module layer, and the output layer, and the overall structure is shown in Figure 1.

The input is a multivariate time series data $X=\left\{x_{\mathrm{it}}\right\}\in {\mathbb{R}}^{N\times T}$ containing T time nodes in N columns, and before being processed layer by layer by the deep neural network, a graph structure $G=\left\{X,W\right\}$ describing the relationship between the input data is first obtained through the smoothing module and the graph building module, where X is the data of each node in the input, and $W_{N\times N}$ is the connection weight matrix between each node. $G=\left\{X,W\right\}$ is fed into the GRGNN module layer and the output layer after several rounds of training and learning to obtain the final prediction result $\hat{X}=\left\{{\hat{X}}_{T+1},{\hat{X}}_{T+2},\ldots ,{\hat{X}}_{T+H}\right\}$ . Where T is the number of time nodes of the input time series data and H is the prediction step size. A mathematical description of the above process can be expressed in Equations 1, 2:


(1) $$G=\mathrm{graphstruct}\left(X\right)$$

(2) $$\left\{{\hat{X}}_{T+1},{\hat{X}}_{T+2},\ldots \ldots ,{\hat{X}}_{T+H}\right\}=F\left(X,G\right)$$

### 2.1 Preprocessing layer

#### 2.1.1 Smoothing processing module

The input data received by the smoothing module are multivariate time series data $\tilde{X}=\left\{{\tilde{x}}_{\mathrm{it}}\right\},i\in {\mathbb{R}}^{N},t\in {\mathbb{R}}^{T}$ . Due to the different statistical rules of the health statistics departments in each country, some countries will postpone the epidemic data from the weekend to Monday of the following week, which is reflected in the data as a line graph with a weekly cycle showing an obvious “sawtooth waveform.” In order to eliminate the negative impact of this problem on the neural network prediction, but also to a certain extent to eliminate some of the noise of the input data, the neural network will be used after the input of a moving window average smoothing processing for a data preprocessing.

The principle of sliding window average smoothing processing is shown in Equation 3, Finally, we will get the smoothed data X after processing the data on day t of the time series will be equal to the average of its data on that day and the data on the n days before it and the n days after it, and $\left(2n+1\right)$ is called the window size. Considering the characteristics of the data in this experiment, n is set to 3, that is, the window size is 7.


(3) $$x_{t}=\frac{1}{2n+1}\overset{t+n}{\underset{i=t-n}{\sum }}{\tilde{x}}_{i},t=\left[n,N-n\right]$$

#### 2.1.2 Graph building blocks

GNN (Graph Neural Network)-based methods need to construct a graph structure $G=\left\{N,E\right\}$ before forecasting multivariate time series. In this study, the number of active cases in a certain geographical area is taken as the object of the study, and the data of each subregion in the geographical area is taken as the node N of the graph, and the edges E of the graph denote the correlation and the magnitude of the influence of each node on each other. In this study, E is represented by the weight matrix $W\in {\mathbb{R}}^{N\times N}$ . The element $w_{ij},i\in \left[0,N-1\right],j\in \left[0,N-1\right]$ in W represents the magnitude of the influence weight of the i th node on the j th node. The graph structure in this study is denoted by $G=\left\{X,W\right\}$ .

Part of the graph structure can be constructed by humans for observation or through experience or knowledge (e.g., road networks in traffic forecasting, grid systems in electrical energy forecasting). However, in general, there is usually no worthwhile sufficient *a priori* experience to accomplish graph construction artificially. For example, in this study, when dealing with data related to epidemics, there may be a situation where the transmission pathways and characteristics of the epidemics under study have yet to be studied, and the existing research and knowledge about them cannot support the construction of the graph. In order to cope with this situation, the correlation between multiple time series is captured in the preprocessing stage through the self-attention mechanism with the GRU (Gated Recurrent Unit) layer before the data is input into the neural network, and the correlation of each time series is determined in a data-driven manner, which then completes the construction of the required graph structure for the neural network (42).

A specific description of the self-attention mechanism approach for the composition layer is given below:

First of all, the multivariate time series $X\in {\mathbb{R}}^{N\times N}$ will be fed into the GRU (Gated Recurrent Unit) layer, which calculates the hidden state corresponding to each time node sequentially. The hidden states corresponding to each time nodes are computed sequentially. Then, we use the last hidden state to calculate the weight matrix through the self-attention mechanism. The mathematical description is as Equation 4–6:


(4) $$W^{Q}=\mathrm{xaviernormal}\left(H\right)$$

(5) $$W^{K}=\mathrm{xaviernormal}\left(H\right)$$

(6) $$\left\{ \begin{array}{l} Q=RWQ\\ K=RWH\\ W=\mathrm{soft}\max\left(\frac{QKT}{\sqrt{d}}\right) \end{array}\right.$$

where Q and K denote the query and key hiding matrices, respectively, and the magnitude of their values are computed by two learnable parameter matrices $W^{Q}$ and $W^{K}$ , respectively, whose initial values are obtained by xavier initialization of the input H (44); d is the size of the dimensions of the two matrices Q and K . The final output adjacency weight square matrix $W\in {\mathbb{R}}^{N\times N}$ will be used with the input multidimensional time series $X\in {\mathbb{R}}^{N\times T}$ , which forms the final graph structure $G=\left\{X,W\right\}$ .

### 2.2 GRGNN layer

**Figure 2:**
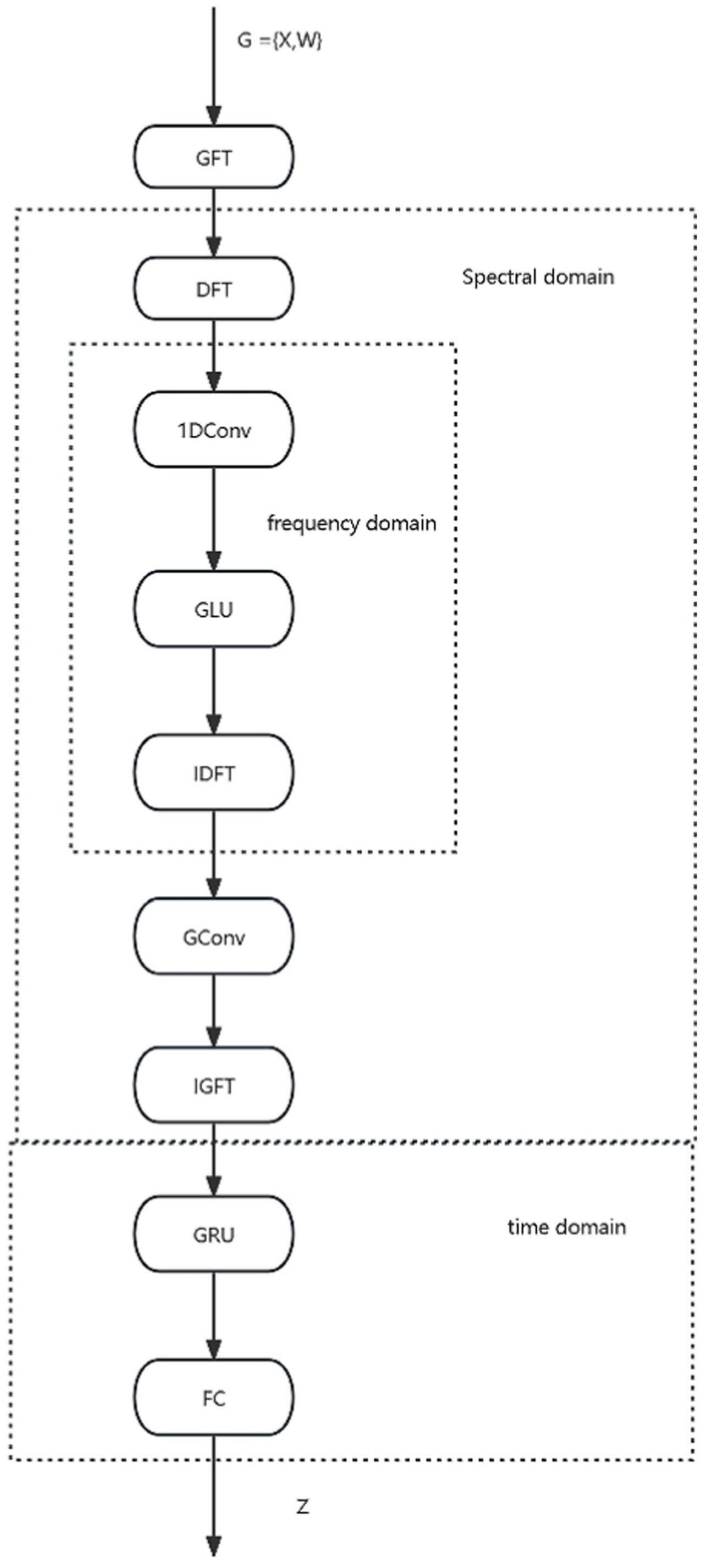
The overall structure of GRGNN module.

The GRGNN layer consists of multiple GRGNN modules stacked in a shortcut connection manner, and the data will be captured and extracted features in the GRGNN modules from the three dimensions of the spectral domain, the frequency domain, and the time domain, respectively. The specific structure of the GRGNN block module, as shown in Figure 2. The features in data will be captured and extracted in three domains of the spectral domain, the frequency domain, and the time domain respectively, in the GRGNN modules. The following is a description of each part of GRGNN block and its functions:

Spectral domain graph convolution is a method that has been widely used in time series forecasting problems. The method has been widely used in time series forecasting problems due to its excellent results in learning potential representations of multiple time series in the spectral domain. The key to the method is the application of the Graph Fourier Transform (GFT) to capture the relationships between time series in the spectral domain. Its output is also a multivariate time series, and the GFT does not explicitly learn the relationship between the data at each time node within a given time series. Therefore, it is necessary to introduce the Discrete Fourier Transform (DFT) to learn the characterization of the input time series in the frequency domain, for example, to capture repetitive features in periodic data.

#### 2.2.1 Frequency domain convolution part

The function of the frequency domain convolution part aims to transfer each individual time series into the frequency domain representation after processing it by DFT, and to learn its features by 1DConv layer in the frequency domain. It consists of four sub-parts in order: discrete Fourier transform (DFT), one-dimensional convolution (1DConv), gated linear unit (GLU), and inverse discrete Fourier transform (IDFT), where DFT and IDFT are used to transform the time series data between time and frequency domains, and 1DConv and GLU are used to learn the features of the time series in the frequency domain. The DFT processing of time sequence usually results in a complex sequence, and the frequency domain convolution is performed on the real part ( ${\hat{X}}_{u}^{r}$ ) and imaginary part ( ${\hat{X}}_{u}^{i}$ ) respectively, and the processing can be expressed by Equation 7 as:


(7) $$\begin{array}{l} M^{*}\left({\hat{X}}_{u}^{*}\right)=GLU\left(\theta_{\tau }^{*}\left({\hat{X}}_{\tau }^{*}\right),\theta_{\tau }^{*}\left({\hat{X}}_{u}^{*}\right)\right)\\ \;=\theta_{\tau }^{*}\left(X_{u}^{*}\right)⊙\sigma^{*}\left(\theta_{\tau }^{*}\left(X_{u}^{*}\right)\right),*\in \left\{r,i\right\} \end{array}$$

Where $\theta_{\tau }^{*}$ denotes the size of the convolution kernel for 1D convolution, ⊙ denotes the Hadamard product operation, and $\sigma^{*}$ denotes the sigmoid activation function. The final result $M^{r}\left({\hat{x}}_{u}^{r}\right)+iM^{i}\left({\hat{x}}_{u}^{i}\right)$ is converted back to the time domain after IDFT processing to participate in the subsequent part of the processing.

#### 2.2.2 Spectral domain graph convolution part

Graph Convolution (29) consists of three parts.

First, Transformation of multivariate time series inputs to the spectral domain via GFT. Second, performing a graph convolution operation on the spectral domain graph structure using a graph convolution operator with a convolution kernel to learn. Third, performing the inverse graph Fourier transform (IGFT) on the spectral domain convolution result to generate the final output.

**Figure 3:**
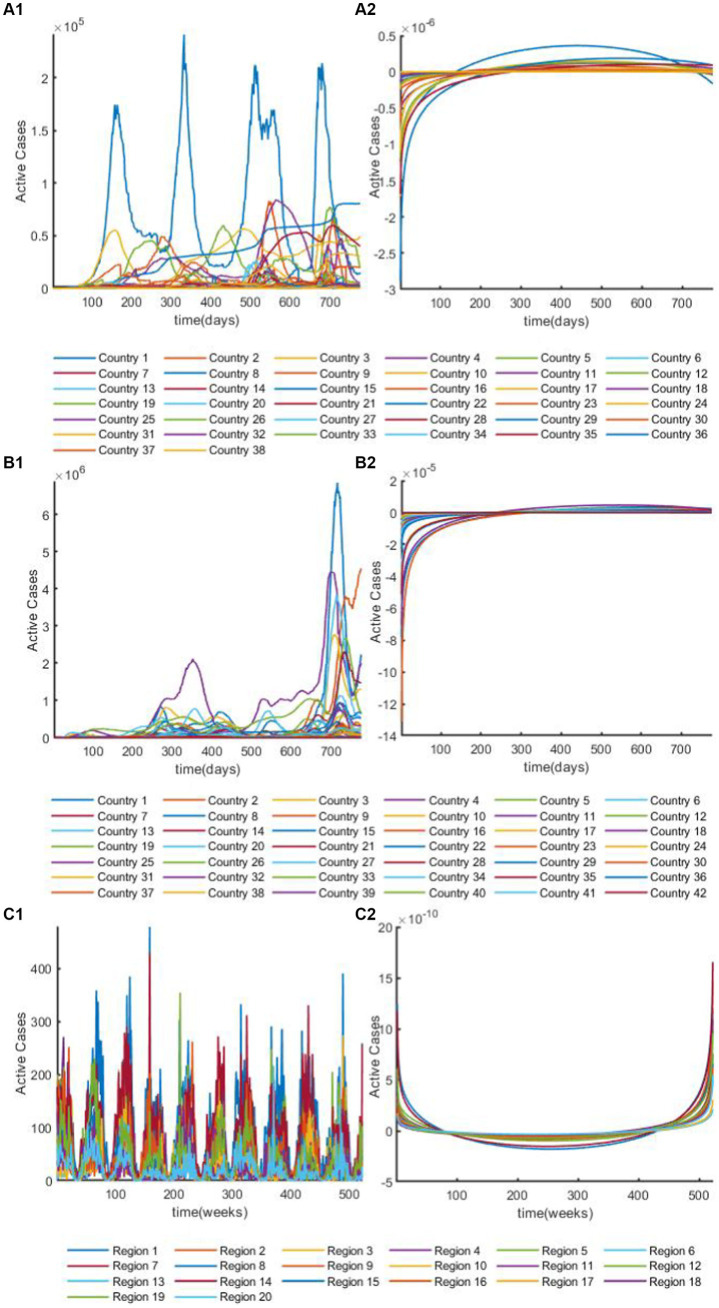
The overview plot of time series after discrete Fourier transform. **(A1)** The overview plot of real parts in time series for African dataset after discrete Fourier transform. **(A2)** The overview plot of image parts in time series for African dataset after discrete Fourier transform. **(B1)** The overview plot of real parts in time series for European dataset after discrete Fourier transform. **(B2)** The overview plot of image parts in time series for European dataset after discrete Fourier transform. **(C1)** The overview plot of real parts in time series for Hungarian dataset after discrete Fourier transform. **(C2)** The overview plot of image parts in time series for Hungarian dataset after discrete Fourier transform.

The graph Fourier transform (GFT) (22) is the basic operator for the convolution of spectral domain graphs. It projects the input graph into a standard orthogonal space where the basis is constructed from the eigenvectors of the normalized graph Laplacian. The normalized graph Laplacian matrix (15) can be computed as follows: $L=I_{N}-D^{-\frac{1}{2}}WD^{-\frac{1}{2}},I_{N}\in {\mathbb{R}}^{N\times N}$ where $I_{N}\in {\mathbb{R}}^{N\times N}$ is the unit matrix and D is the degree matrix with diagonal element $D_{ii}=\underset{j}{\sum }W_{ij}$ . Then, the eigenvalue decomposition of the Laplace matrix is performed to obtain $L=U\Lambda U^{T}$ , where $U\in {\mathbb{R}}^{N\times N}$ is the matrix of eigenvectors and Λ is the diagonal matrix of eigenvalues. After, the GFT, time series will be transformed into complex numbers, for example, three datasets after DFT are shown in Figure 3. For a detailed introduction to the dataset, see section 2.4.1. Given a multivariate time series $X\in {\mathbb{R}}^{N\times T}$ , the GFT and IGFT operators and specific operations are, respectively, denoted as $GF\left(X\right)=U^{T}X=\hat{X}$ and $GF^{-1}\left(\hat{X}\right)=U\hat{X}$ . The graph convolution operator is realized as a function $g_{\Theta }\left(\Lambda \right)$ of the eigenvalue matrix Λ , where Θ is the convolution kernel parameter.

#### 2.2.3 Time domain GRU (gated recurrent units) layer

Recurrent Neural Networks (RNN) are a type of neural networks with an inner recurrent loop structure (23). The reformed GRGNN with its introduction and GRGNN’s application on the epidemic field is an important innovation in this study. GRU (Gated Recurrent Unit) processes sequences by traversing the sequence elements and generating a hidden state that contains pattern information related to the historical data, which contains the before-and-after relationships of the sequences. GRUs (Gated Recurrent Units) (23) are a type of recurrent neural networks in which each loop unit adaptively captures dependencies at different time scales. Similar to LSTM (Long Short-Term Memory) units, GRUs (Gated Recurrent Units) have a gating unit that regulates the information within the unit, but do not have a separate storage unit like LSTM (Long Short-Term Memory).


(8) $$z_{t}=\sigma \left(W_{z}\;\cdot \;\left[h_{t-1},x_{t}\right]\right)$$

(9) $$r_{t}=\sigma \left(W_{r}\;\cdot \;\left[h_{t-1},x_{t}\right]\right)$$

(10) $${\tilde{h}}_{t}=\tanh\left(W\left[r_{t}\;\cdot \;h_{t-1},x_{t}\right]\right)$$

(11) $$h_{t}=\left(1-z_{t}\right)\;\cdot \;h_{t-1}+z_{t}\cdot {\tilde{h}}_{t}$$

The specific mathematical description of GRU (Gated Recurrent Unit) is shown in Equation 8–11, there are only two gate units in GRU (Gated Recurrent Unit), one is reset gate and the other is update gate, and the role of reset gate is similar to that of input gate and forgetting gate in LSTM (Long Short-Term Memory), $\left(1-z\right)$ is equivalent to the input gate, and z is equivalent to the forgetting gate. The GRU (Gated Recurrent Unit) method uses fewer threshold units to accomplish a similar task as the LSTM (Long Short-Term Memory) method, so the GRU (Gated Recurrent Unit) method is usually considered when there is a lack of computational power or a desire to improve the training speed and efficiency of neural network learning. The GRU (Gated Recurrent Unit) method uses fewer gate units than the LSTM (Long Short-Term Memory) method and accomplishes a similar task.

### 2.3 Implementation and parameter design

The GRGNN method was developed using the Python language based on Pytorch and MATLAB language, the experiments of GRGNN were performed on a deep-learning server with NVIDIA Quadro GV100L GPU *1, Intel Xeon Gold 6,138 CPU *1 and DDR4 32G RAM *8, the operation system of Ubuntu 18.04.6 LTS. The baseline methods were all implemented using MATLAB language. on clearance version.

Hyperparameters such as input length, learning rate, batch size, training time and number of hidden units needed to be set in the GRGNN. Empirically, normalization method was set to z-score, input length to 15, learning rate to 4.7e-4, batch size to 15 and training epoch to 150 and the number of layers to 7. Additionally, the ADAM optimizer was used in the training process.

### 2.4 Dataset, baseline methods and evaluation indicators

#### 2.4.1 Datasets

**Figure 4:**
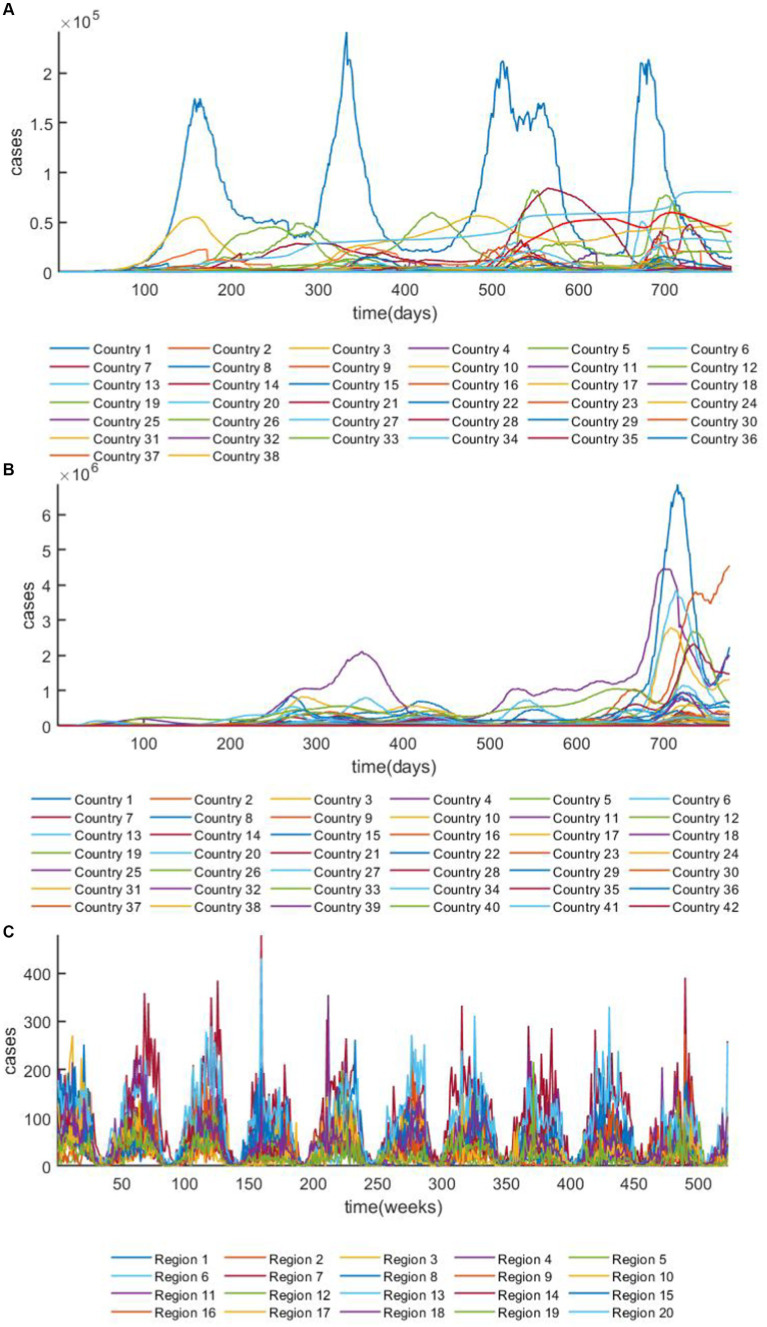
The overview plot of the datasets. **(A)** The overview plot of ARMSE of the 38 African countries’ COVID-19 dataset. **(B)** The overview plot of the 42 European countries’ COVID-19 dataset. **(C)** The overview plot of 20 Hungarian regions’ chickenpox dataset.

In this study, the prediction effect of GRGNN was tested using the 42 European countries’ COVID-19 dataset, the 38 African countries’ COVID-19 dataset and the 20 Hungarian regions’ chickenpox dataset, the overview plots of the datasets are shown in Figure 4 both COVID-19 datasets in this study were collected from publicly available data provided by the Worldometers website (45). Worldometer is run by an international team of developers, researchers, and volunteers with the goal of making world statistics available in a thought-provoking and time relevant format to a wide audience around the world Government’s communication channels which makes the data from it more reliable and realistic. The 42 European countries’ COVID-19 dataset contains 42 time series, and the length of each time series in the dataset is 776. The 38 African countries’ COVID-19 dataset contains 38 time series, and the length of each time series in the dataset is 776. The 20 Hungarian regions’ chickenpox dataset contains 20 time series, and the length of each time series in the dataset is 523. Two COVID-19 datasets analyzed during the current study are available in the [Worldometers] repository.^1^ The daily active case count data of each country were collected for a total of 776 days from February 15, 2020 to April 1, 2022, and the data were cleaned to exclude from the data that existed for more than 20 days without updating the data, and the data that had a negative number of active cases or other statistical errors, finally we classify the data that met the above requirements to obtain the continental active case dataset. The 20 Hungarian regions’ chickenpox dataset was chosen to collect weekly chickenpox diagnosis data from 20 regions in Hungary for 523 weeks from January 3, 2005 to December 29, 2014. The 20 Hungarian regions’ chickenpox dataset are available,^2^ the dataset was downloaded from Kaggle (46), a website that focuses on providing developers and data scientists with a platform to hold machine learning competitions, host databases, and write and share code. The Hungarian chickenpox dataset, as a typical multivariate time series prediction problem dataset was consisted by the time series collected from the Hungarian Epidemiological Info, a weekly bulletin of morbidity and mortality of infectious disease in Hungary. This dataset was tested on the Kaggle platform with many time series prediction methods and data visualization methods.

#### 2.4.2 Baseline methods

Three widely used neural network architectures; LSTM (Long Short-Term Memory), GRU (Gated Recurrent Unit), CNN-LSTM and a statistical method, were chosen as the control group in this study, the statistical methods include, weighted moving average method(WMA) (47), Gaussian function method (48) and polynomial functions method (48):

The following 7 baseline methods were used to compare the performance with the GRGNN:

ARIMA (15): ARIMA (Autoregressive Integrated Moving Average Model) is a widely applied time series forecasting method, extensively used across various fields. This paper adopts it as a classical statistical prediction method to compare with machine learning approaches for forecasting COVID-19 data in Africa. Its specific definition is given in Equation 12.


(12) $$\left(1-\overset{p}{\underset{i=1}{\sum }}\varphi_{i}L^{i}\right){\left(1-L\right)}^{d}X_{i}=\left(1+\overset{q}{\underset{i=1}{\sum }}\theta_{i}L^{i}\right)\varepsilon_{t}$$

Herein, L represents the lag operator, with d>0,d∈Z . The main steps of this method are as follows:

The prediction will finish in 4 steps: step 1, Time series preprocessing. The primary purpose here is to make the input to the ARIMA model a stationary time series. If the data series is non-stationary and exhibits certain growth or decline trends, it is necessary to differentiate the data. Step 2, Establishing the model based on identification rules for time series models. If the partial autocorrelation function of the stationary series is truncated while the autocorrelation function is tailed, the series is suitable for an AR model; if the partial autocorrelation function is tailed while the autocorrelation function is truncated, the series is suitable for an MA model; if both the partial autocorrelation and autocorrelation functions are tailed, the series fits an ARIMA model. Step 3, Determining the order of AR and MA. Utilize the Akaike Information Criterion (AIC) and Bayesian Information Criterion (BIC) to determine the orders p and q of AR and MA, respectively. Step 4, ARIMA fitting and forecasting. Fit the ARIMA model, then use the fitted results to forecast the test set. It’s worth mentioning that these results are after one differentiation, and the forecasted values need to be restored through inverse differentiation.

weighted moving average method (WMA) (47): the weighted moving average (WMA) method is a time series analysis technique that assigns different weights to historical observations based on their relative importance. Unlike the simple moving average (SMA) method, which assigns equal weight to all observations, the WMA method seeks to accentuate the impact of more recent data and reduce the impact of older data points. The WMA method calculates the weighted average of a sequence of observations, with the most recent values carrying the highest weightings. The weightings assigned to each observation are typically determined by a predefined set of coefficients or by subjective judgment based on the characteristics of the data being analyzed. The WMA method is frequently used in financial market analysis to identify trends and forecast future prices. The specific definition of WMA is given in Equation 13.


(13) $${\hat{X}}_{t+1}=\omega_{0}X_{t}+\omega_{1}X_{t-1}+\cdots +\omega_{N}X_{t-N+1}$$

Where ${\hat{X}}_{t+1}$ denotes the prediction for the time point t+1 , $X_{*}$ stands for the observation value, and $\omega_{*}$ stands for the weight of $X_{*}$ .

Gaussian function fitting method (48): one of the most popular curve fitting algorithms for fitting the time series with a n-order Gaussian function $G\left(x\right)$ , which has been widely applied in prediction. The specific definition of Gaussian function fitting method is given in Equation 14. In this research we applied 3-order Gaussian function to fitting each time series.


(14) $$G\left(x\right)=a_{1}\cdot e^{-{\left(\frac{x-b_{1}}{c_{1}}\right)}^{2}}+a_{2}\cdot e^{-{\left(\frac{x-b_{2}}{c_{2}}\right)}^{2}}+a_{3}\cdot e^{-{\left(\frac{x-b_{3}}{c_{3}}\right)}^{2}}$$

Polynomial function fitting method (48): one of the most popular curve fitting algorithms for fitting the time series with a n-order polynomial function, which has been widely applied in prediction. The specific definition of polynomial function fitting method is given in Equation 15. in this research we applied 5-order polynomial function $G\left(x\right)$ to fitting each time series.


(15) $$G\left(x\right)=p_{1}\cdot x^{5}+p_{2}\cdot x^{4}+p_{3}\cdot x^{3}+p_{4}\cdot x^{2}+p_{5}\cdot x+p_{6}$$

LSTM (Long Short-Term Memory): Long Short-Term Memory networks were first introduced by Hochreiter in 1997 (22). They are a specific form of RNN (Recurrent Neural Network), which is a general term for a series of neural networks that can process sequential data.

Generally, RNNs possess three characteristics: first, they can generate an output at each time step, with connections between hidden units being cyclic; second, they produce an output at each time step, where the output at a given time step is only cyclically connected to the hidden unit of the next time step; third, RNNs contain hidden units with cyclic connections and can process sequential data to produce a single prediction.

LSTM (Long Short-Term Memory) is such a gated RNN. The ingenuity of LSTM (Long Short-Term Memory) lies in the addition of input, forget, and output gates, allowing the self-recurrent weights to vary. Thus, the integration scale at different moments can dynamically change even when the model parameters are fixed, thereby avoiding problems of gradient vanishing or exploding.

Each LSTM (Long Short-Term Memory) unit is composed of a memory cell and three gating units: the input gate, the output gate, and the forget gate. Within this architecture, LSTM (Long Short-Term Memory) attempts to create a controlled flow of information by deciding what information to “forget” and what to “remember,” thereby learning long-term dependencies.


(16) $$z_{t}=\sigma \left(W_{z}\cdot \left[h_{t-1},x_{t}\right]\right)$$

(17) $$f_{t}=\sigma \left(U_{g}x_{t}+W_{g}h_{t-1}+b_{g}\right)$$

(18) $${\tilde{c}}_{t}=\tanh\left(U_{c}x_{t}+W_{c}h_{t-1}+b_{c}\right)$$

(19) $$c_{t}=g_{t}⊙c_{t-1}+i_{t}⊙{\tilde{c}}_{t}$$

(20) $$o_{t}=\sigma \left(U_{o}x_{t}+W_{o}h_{t-1}+b_{o}\right)$$

(21) $$h_{t}=o_{t}⊙\tanh\left(c_{t}\right)$$

More specifically, the input gate $i_{t}$ alongside the second gate ${\tilde{c}}_{t}$ control the new information stored in the memory state $c_{t}$ at a certain time t . The forget gate $f_{t}$ controls the disappearance or retention of information from time t−1 in the storage unit, while the output gate $o_{t}$ controls which information can be outputted by the storage unit. Equation 16–21 succinctly describe the operations performed by an LSTM (Long Short-Term Memory) unit.

Herein, $x_{t}$ represents the input at a certain moment, $W_{*}$ and $U_{*}$ represent weight matrices, $b_{*}$ denotes the bias vector, σ is the sigmoid function, and the operator ⊙ represents element-wise multiplication. Finally, the hidden state unit $h_{t}$ , which forms part of the memory cell’s output, is calculated as shown in Equation 21.

It is noteworthy that if multiple LSTM (Long Short-Term Memory) layers are stacked together, the memory state $c_{t}$ and hidden state $h_{t}$ of each LSTM (Long Short-Term Memory) layer will serve as inputs to the next LSTM (Long Short-Term Memory) layer.

In this paper, the main hyperparameters for the LSTM (Long Short-Term Memory) method are set as follows: the number of iterations is 150, the number of hidden units is 400, the initial learning rate is 0.001, and the optimizer used is ADAM.

GRU (Gated Recurrent Unit): The GRU (Gated Recurrent Unit) is also a type of recurrent neural network. Like LSTM (Long Short-Term Memory), it was developed to address issues related to long-term memory and gradients in backpropagation. Compared to LSTM (Long Short-Term Memory), using GRU (Gated Recurrent Unit) can achieve comparable results and is easier to train, significantly enhancing training efficiency. Therefore, GRU (Gated Recurrent Unit) is often preferred, especially in scenarios with limited computational power or when there is a need to conserve computational resources.

GRU (Gated Recurrent Unit) has only two gating units: a reset gate and an update gate, as shown in Equation 8–11, where $x_{t}$ represents the input at a given time, $W_{*}$ represents a weight matrix, σ denotes the tanh function, $z_{t}$ is the state of the update gate, and $r_{t}$ is the reset gate. The function of the reset gate is similar to the input and forget gates in LSTM (Long Short-Term Memory), where $1-z_{t}$ acts like the input gate, and $z_{t}$ functions as the forget gate. Given that GRU (Gated Recurrent Unit) uses fewer gating units to accomplish tasks similar to those of LSTM (Long Short-Term Memory), GRU (Gated Recurrent Unit) is typically considered in situations where computational capacity is limited.

In this paper, the hyper parameters for the GRU (Gated Recurrent Unit) method are set as follows: the number of maximum training epoch is 150, the batch size is 12, the number of hidden units is 400, the initial learning rate is 0.001, and the optimizer used is ADAM.

CNN-LSTM: CNN-LSTM is an advanced neural network architecture that combines Convolutional Neural Networks (CNNs) and LSTMs (Long Short-Term Memory networks) to harness the strengths of both in processing sequential data. This hybrid model is particularly effective for tasks where the input data involves both spatial and temporal dimensions, making it popular in areas such as video analysis, natural language processing, and time series forecasting.

Crucially, to adapt the time series data for the CNN-LSTM architecture, we employ lag features transformation. This involves creating new datasets where each feature corresponds to the original data shifted by values within a specified lag range, effectively capturing temporal dependencies across multiple time steps. These transformed datasets are then organized into matrices, with each column representing a different lagged version of the data, making it suitable for sequential processing by the model.

For the LSTM (Long Short-Term Memory) component, it is the same like the LSTM (Long Short-Term Memory) methods we introduced above. And for the CNN component, the data is initially processed through a sequence folding layer, transforming the sequential input into a format amenable to convolutional operations. This step is pivotal for extracting spatial features from the lagged inputs, which are then unfolded and flattened to preserve the temporal sequence structure, allowing the subsequent LSTM (Long Short-Term Memory) layers to learn long-term dependencies from these extracted features effectively. By meticulously mapping our datasets through these preparatory stages, we ensure that the CNN-LSTM architecture leverages both spatial and temporal dimensions of the data, thereby enhancing the model’s forecasting accuracy.

In this paper, the hyper parameters for the CNN-LSTM method are set as follows: the number of maximum training epoch is 150, the batch size is 12, the lag is 8, the number of hidden units [LSTM (Long Short-Term Memory) component] is 150, the initial learning rate is 0.001, and the optimizer used is ADAM.

#### 2.4.3 Evaluation indicators

Average RMSE and average MAE are used as evaluation metrics to measure the magnitude of error in the prediction results:

The average RMSE is calculated by sequentially calculating the RMSE for each of the N countries in the prediction result of the sequence prediction step H . The specific mathematical description is as following Equation 22:


(22) $$\left\{ \begin{array}{l} RMSE_{i}=\sqrt{\frac{\sum_{i=1}^{H}y_{\mathrm{pred},i}-y_{obs,i}}{H}}\\ RMSE_{ave}=\frac{\sum_{i=1}^{N}RMSE_{i}}{N} \end{array}\right.$$

The average MAE is calculated by sequentially calculating the MAE for each of the N countries in the prediction result of the sequence prediction step H , and then calculating the average value, which is mathematically described as following Equation 23:


(23) $$\left\{ \begin{array}{l} MAE_{i}=\frac{\sum_{i=1}^{H}|y_{\mathrm{pred},i}-y_{obs,i}|}{H}\\ MAE_{ave}=\frac{\sum_{i=1}^{N}MAE_{i}}{N} \end{array}\right.$$

## 3 Results

Predictions were made using GRGNN, LSTM (Long Short-Term Memory), GRU (Gated Recurrent Unit), CNN-LSTM, and ARIMA for 42 countries in Europe, 38 countries in Africa, two continents’ COVID-19 active case datasets, and Hungary’s 20 regions’ varicella datasets, respectively. The last 2 weeks (14 days), 3 weeks (21 days), 4 weeks (28 days), 5 weeks (35 days), and 6 weeks (42 days) data were taken as the test set in the prediction, and after dividing the test set, all the data prior to the test set data were divided into the training set and validation set in the ratio of 10:1.

The prediction results of each method for each dataset at different step sizes are shown in Tables 1–5.

**Table 1:** Prediction results for each prediction method for each dataset for 2 weeks (14 days).

	African dataset		European dataset		Hungarian dataset	
	ARMSE	AMAE	ARMSE	AMAE	ARMSE	AMAE
GRGNN	683.27	621.38	54568.57	49345.78	28.82	23.64
LSTM	1288.20	1071.58	78093.59	64940.05	29.69	24.57
CNN-LSTM	812.45	790.14	38421.52	31634.68	32.85	26.29
GRU	1115.73	907.52	56406.04	47197.14	32.21	27.66
ARIMA	783.04	657.50	40086.60	42310.69	29.61	23.83
Poly	4620.15	4480.89	301141.17	298245.73	44.11	36.52
Gauss	2289.51	2214.87	109168.62	103422.19	41.55	34.48
WMA	820.68	691.10	70424.89	62469.22	35.13	29.24

**Table 2:** Prediction results for each prediction method for each dataset for 3 weeks (21 days).

	African dataset		European dataset		Hungarian dataset	
	ARMSE	AMAE	ARMSE	AMAE	ARMSE	AMAE
GRGNN	836.26	770.61	75623.18	83044.94	31.47	28.47
LSTM	1375.33	1116.70	113619.62	135365.11	33.62	28.75
CNN-LSTM	915.06	892.35	48978.62	55363.46	35.65	31.46
GRU	1608.06	1260.72	115653.55	144957.77	34.41	28.90
ARIMA	997.03	848.51	68989.77	82938.58	35.22	29.77
Poly	5428.18	5195.21	409270.15	401718.39	29.61	28.93
Gauss	2641.67	2531.15	188754.28	181754.93	36.31	28.60
WMA	1007.55	831.20	119667.10	104058.45	29.70	24.21

**Table 3:** Prediction results for each prediction method for each dataset for 4 weeks (28 days).

	African dataset		European dataset		Hungarian dataset	
	ARMSE	AMAE	ARMSE	AMAE	ARMSE	AMAE
GRGNN	748.42	858.05	111743.17	123580.61	27.48	21.75
LSTM	2296.05	2775.97	125159.58	151888.46	28.02	22.36
CNN-LSTM	882.52	921.98	88773.80	97859.95	29.10	22.21
GRU	1718.08	2188.22	188863.87	161955.56	28.88	23.04
ARIMA	921.32	1082.60	136034.82	112387.49	27.55	20.98
Poly	6628.94	6351.37	534460.56	520915.42	35.01	26.59
Gauss	3254.86	3078.51	214257.05	194298.74	33.07	25.51
WMA	1437.04	1228.77	152546.44	132098.87	28.13	22.02

**Table 4:** Prediction results for each prediction method for each dataset for 5 weeks (35 days).

	African dataset		European dataset		Hungarian dataset	
	ARMSE	AMAE	ARMSE	AMAE	ARMSE	AMAE
GRGNN	820.70	1004.61	120230.14	127749.64	27.27	21.45
LSTM	2507.72	3072.21	255698.55	219314.79	27.40	21.55
CNN-LSTM	1536.70	1593.65	128824.08	111020.42	28.14	21.91
GRU	2234.65	2667.76	250900.80	213550.93	28.26	22.00
ARIMA	1537.67	1731.40	150250.91	125333.12	29.96	22.37
Poly	8436.73	8093.56	652543.68	625536.10	34.23	26.48
Gauss	4526.36	4227.41	212263.48	193738.61	32.04	25.00
WMA	2525.29	2301.08	238699.26	209711.46	30.51	22.38

**Table 5:** Prediction results for each prediction method for each dataset for 6 weeks (42 days).

	African dataset		European dataset		Hungarian dataset	
	ARMSE	AMAE	ARMSE	AMAE	ARMSE	AMAE
GRGNN	1545.62	1763.28	124665.83	133453.75	25.51	19.17
LSTM	3418.20	4090.81	308407.33	367230.08	27.58	22.07
CNN-LSTM	1657.79	1810.84	124829.94	153435.48	26.18	20.08
GRU	4648.19	5709.85	232157.67	269820.41	25.72	20.48
ARIMA	2673.66	3035.86	188922.10	229932.70	27.45	20.27
Poly	10843.29	10305.87	739501.24	697691.22	33.02	24.93
Gauss	4735.57	4382.75	251950.31	218247.07	30.33	23.09
WMA	3435.84	3093.05	426603.38	363924.52	27.98	20.14

As can be seen from Table 1, with a prediction step of 2 weeks (14 days), GRGNN achieves optimal results for both the African and Hungarian datasets, and slightly underperforms the CNN-LSTM method and the ARIMA method for the European dataset. The LSTM (Long Short-Term Memory) method and the GRU (Gated Recurrent Unit) method underperform in all datasets. The CNN-LSTM method performs best in the prediction of the European dataset, and underperforms GRGNN and ARIMA in the African dataset, and performs worse in the Hungarian dataset. The ARIMA method has the best prediction accuracy of the eight methods. The CNN-LSTM method performs best in the prediction of the European dataset, while it does not perform as well as GRGNN and ARIMA on the African dataset, and performs even worse on the Hungarian dataset. The prediction accuracy of the ARIMA method is in the middle of the range of the eight methods. The WMA method can achieve predictions with an accuracy approximately equal to that of ARIMA. Conversely, the Gaussian function method and the polynomial function method produce predictions significantly deviating from the real data, obtaining the lowest accuracies among all eight methods across all three datasets.

As can be seen from Table 2, the comparison of the overall prediction results when extending the prediction step to 3 weeks (21 days) is not much different from that of the prediction step of 2 weeks. The GRGNN method still achieves the best results in the prediction of both the African and Hungarian datasets, and is slightly less accurate in the prediction of the European dataset than the CNN-LSTM and the ARIMA methods. The prediction accuracy of the LSTM (Long Short-Term Memory) method and the GRU (Gated Recurrent Unit) method is the worst two of the eight methods in the African and European datasets. The prediction errors of LSTM (Long Short-Term Memory) and GRU (Gated Recurrent Unit) methods in the African and European datasets are the worst two out of the eight methods. The CNN-LSTM method still performs the best in the prediction of the European dataset. The ARIMA method does not achieve the optimal prediction accuracy but outperforms LSTM (Long Short-Term Memory) and GRU (Gated Recurrent Unit) in the African and European datasets, and outperforms CNN-LSTM in the Hungarian dataset in terms of prediction error. The WMA method still yields slightly inferior results compared to ARIMA and marginally better outcomes than the LSTM (Long Short-Term Memory) method. However, the Gaussian function method and the polynomial function method continue to exhibit the poorest two results.

As can be seen from Table 3, with a prediction step of 4 weeks (28 days), GRGNN still maintains the optimal prediction in the prediction of the African and Hungarian datasets, and the prediction results in the European dataset are only slightly inferior to those of the CNN-LSTM method. The prediction errors of the LSTM (Long Short-Term Memory) method and the GRU (Gated Recurrent Unit) method are still poor in the African and European datasets. The CNN-LSTM method still performs optimally in the prediction of the European dataset, but poorly in the European dataset. The ARIMA method is still in the mid-range of the eight prediction mid-range levels. Still performs the best in prediction, but has poor prediction in the Hungarian dataset. The prediction accuracy of the ARIMA method is still in the middle of the range of the 5 prediction mid-range. The performance of the WMA method is slightly inferior to the ARIMA method but slightly superior to the GRU (Gated Recurrent Unit) and LSTM (Long Short-Term Memory) methods. However, the Gaussian method and the polynomial method remain the least effective, exhibiting significant errors in their prediction results.

As can be seen from Table 4, when the prediction step size is set to 5 weeks (35 days), the ranking of the prediction results of each method is not much different from that of the case with a step size of 4 weeks, and it is worth noting that: the main change occurs in the prediction results for European data, and the average index of GRGNN exceeds that of CNN-LSTM as the smallest among the prediction methods. The performance of the WMA method deteriorates rapidly, reaching a point where it only outperforms two other methods. The Gaussian function method and the polynomial function method still remain the poorest performers, with their accuracy indices worsening even further as the prediction steps increase.

As can be seen from Table 5, when the prediction step size is 6 weeks (42 days), the average of the prediction results of GRGNN in the prediction of the European dataset exceeds that of the CNN-LSTM (Long Short-Term Memory) method to become the smallest among the results of each prediction method, and realizes the prediction accuracy of the prediction of each data to be the highest among all eight prediction methods. The prediction error of WMA only slightly exceeds that of LSTM (Long Short-Term Memory) and GRU (Gated Recurrent Unit), placing its results ahead of both LSTM (Long Short-Term Memory) and GRU (Gated Recurrent Unit). However, it falls short compared to GRGNN, CNN-LSTM, and ARIMA methods. The polynomial method and Gaussian function method persist as the least effective, exhibiting the highest ARMSE and AMAE values.

**Figure 5:**
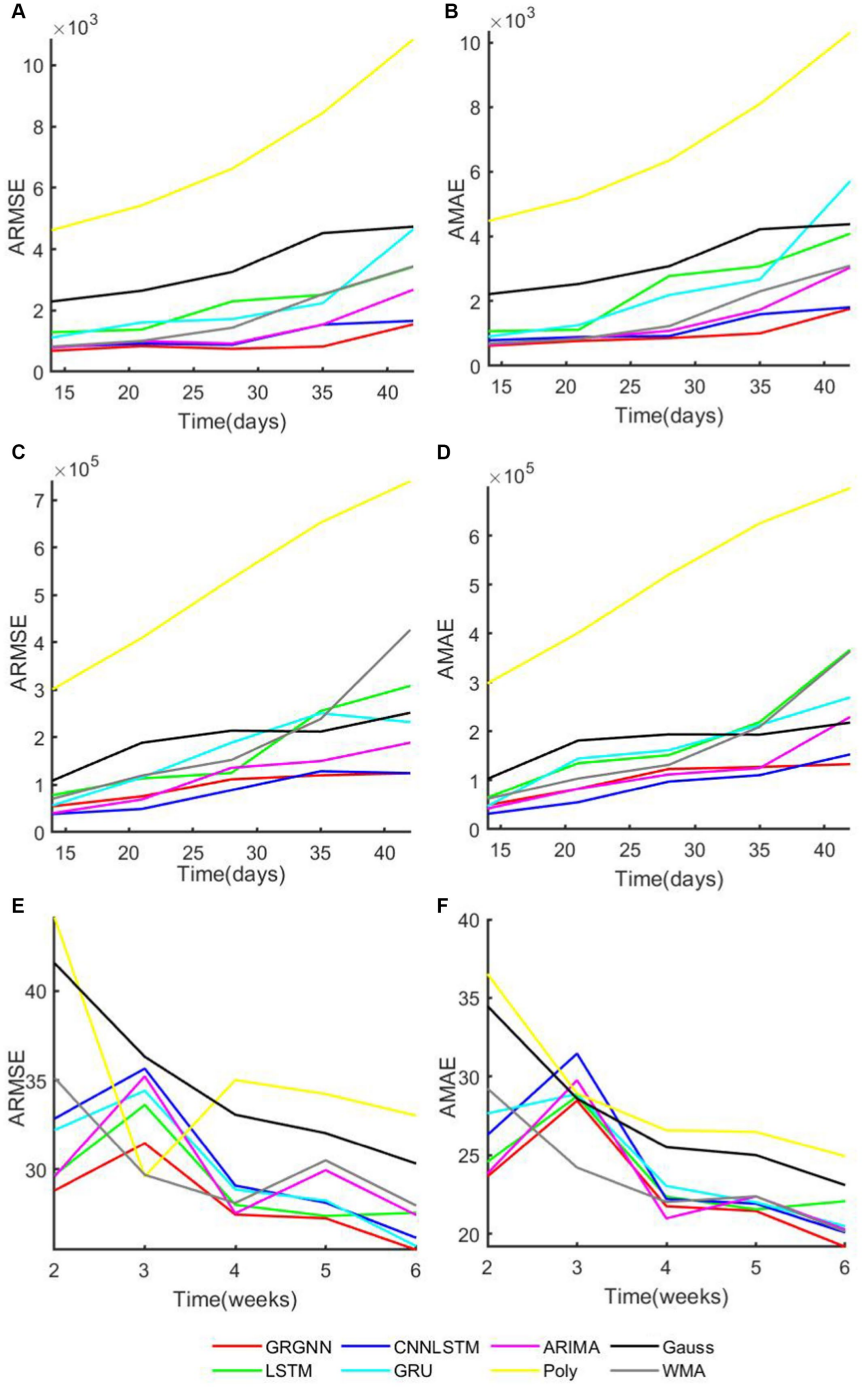
The overview plot of evaluation indicator of datasets **(A)** the overview plot of ARMSE of the 38 African countries’ COVID-19 dataset. **(B)** The overview plot of AMAE of the 38 African countries’ COVID-19 dataset. **(C)** the overview plot of ARMSE of the 42 European countries’ COVID-19 dataset. **(D)** The overview plot of AMAE of the 42 European countries’ COVID-19 dataset. **(E)** the overview plot of ARMSE of the20 Hungarian regions’ Chickenpox dataset. **(F)** The overview plot of AMAE of the 20 Hungarian regions’ Chickenpox dataset.

The average indictors of the prediction results of each method in each dataset are plotted at different step sizes, as shown in Figure 5.

**Figure 6:**
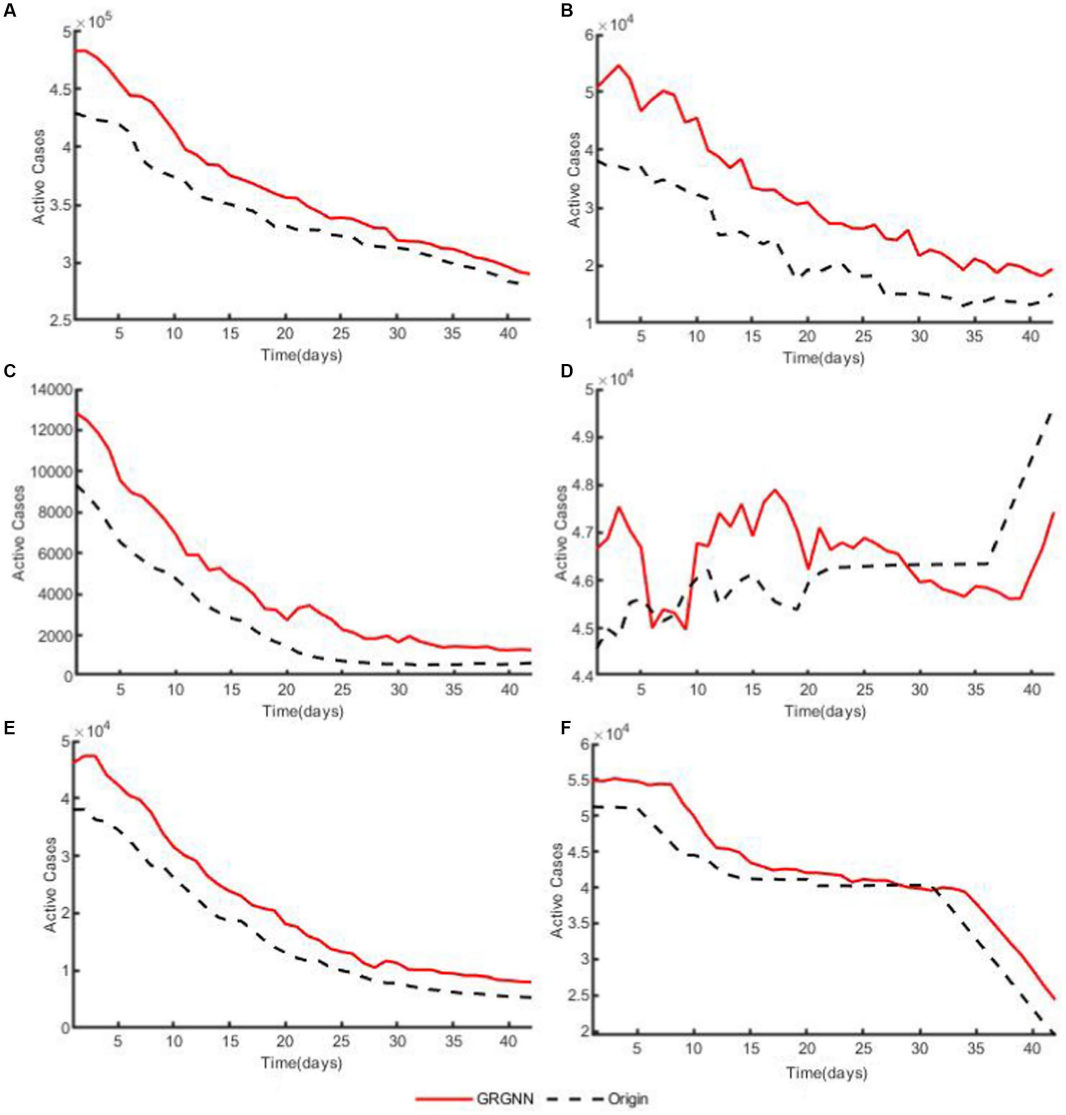
The plots of original data and prediction result for countries from the 38 African countries’ COVID-19 dataset of GRGNN. **(A)** The plot of original data and prediction result for the total cases of the 38 African countries’ COVID-19 dataset of GRGNN. **(B)** The plot of original data and prediction result for Country1 from the 38 African countries’ COVID-19 dataset of GRGNN. **(C)** The plot of original data and prediction result for County2 from the 38 African countries’ COVID-19 dataset of GRGNN. **(D)** The plot of original data and prediction result for Country3 from the 38 African countries’ COVID-19 dataset of GRGNN. **(E)** The plot of original data and prediction result for Country4 from the 38 African countries’ COVID-19 dataset of GRGNN. **(F)** The plot of original data and prediction result for Country5 from the 38 African countries’ COVID-19 dataset of GRGNN.

**Figure 7:**
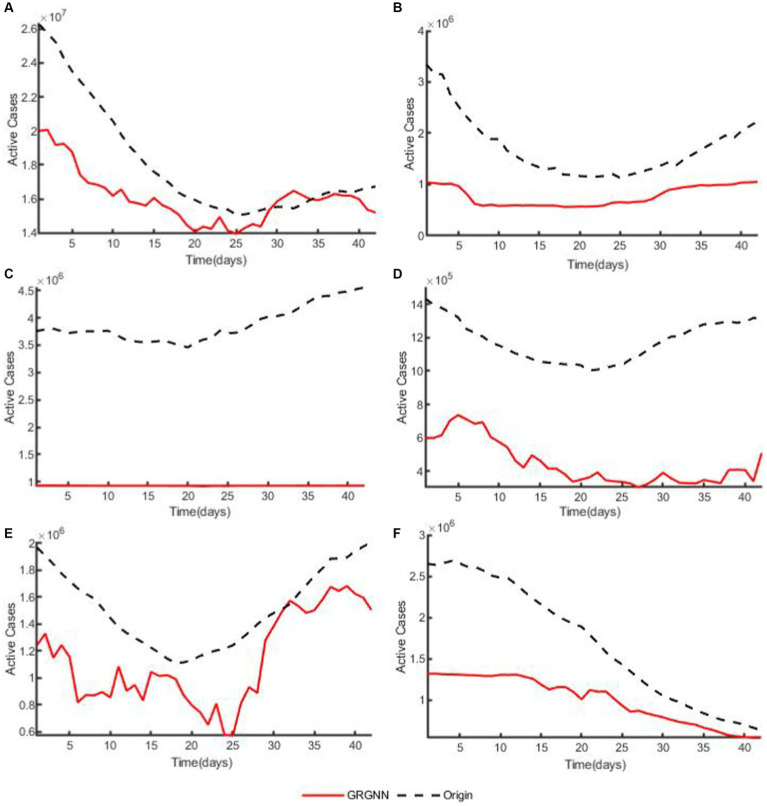
The plots of original data and prediction result for countries from the 42 European countries’ COVID-19 dataset of GRGNN. **(A)** The plot of original data and prediction result for the total cases of the 42 European countries’ COVID-19 dataset of GRGNN. **(B)** The plot of original data and prediction result for Country1 from the 42 European countries’ COVID-19 dataset of GRGNN. **(C)** The plot of original data and prediction result for County2 from the 42 European countries’ COVID-19 dataset of GRGNN. **(D)** The plot of original data and prediction result for Country3 from the 42 European countries’ COVID-19 dataset of GRGNN. **(E)** The plot of original data and prediction result for Country4 from the 42 European countries’ COVID-19 dataset of GRGNN. **(F)** The plot of original data and prediction result for Country5 from the 42 European countries’ COVID-19 dataset of GRGNN.

**Figure 8:**
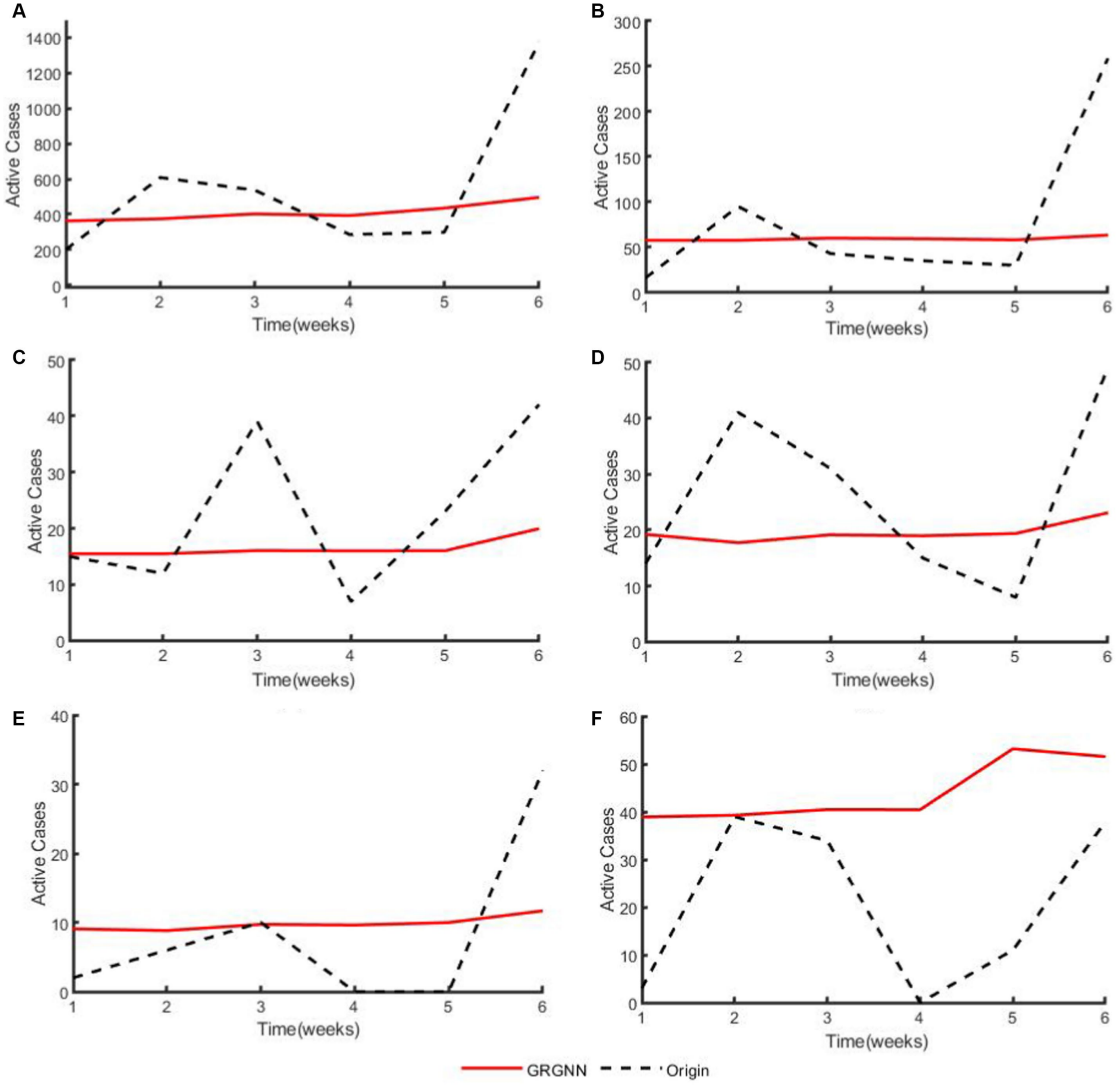
The plots of original data and prediction result for regions from the 20 Hungarian regions’ Chickenpox dataset of GRGNN. **(A)** The plot of original data and prediction result for the total cases of the 20 Hungarian regions’ Chickenpox dataset of GRGNN. **(B)** The plot of original data and prediction result for Region1 from the 20 Hungarian regions’ Chickenpox dataset of GRGNN. **(C)** The plot of original data and prediction result for Region2 from the 20 Hungarian regions’ Chickenpox dataset of GRGNN. **(D)** The plot of original data and prediction result for Region3 from the 20 Hungarian regions’ Chickenpox dataset of GRGNN. **(E)** The plot of original data and prediction result for Region4 from the 20 Hungarian regions’ Chickenpox dataset of GRGNN. **(F)** The plot of original data and prediction result for Region5 from the 20 Hungarian regions’ Chickenpox dataset of GRGNN.

To enhance the clarity and simplicity of conveying the prediction results, we have selected 5 time series from each dataset, focusing on a prediction step set to 6 weeks (42 days) for visualization. Specifically, we depict the time series data of 5 countries from the 38 African countries’ COVID-19 dataset in Figure 6, and the time series of 5 countries from the 42 European countries’ COVID-19 dataset in Figure 7, and illustrate the time series of 5 regions from the 20 Hungarian regions’ chickenpox dataset in Figure 8. Through these figures, it becomes evident that GRGNN generally captures and mirrors the trends observed in the majority of the time series from the original real-world data.

## 4 Discussion

Observing Tables 1–5, it can be found that for the prediction results of the data of the 38 African countries’ COVID-19 dataset and the 20 Hungarian regions’ chickenpox dataset, GRGNN is able to achieve better prediction results compared with other prediction methods at different prediction steps, and the average RMSE and average MAE of its prediction results are the smallest among the prediction methods at different steps, which indicates that GRGNN is able to capture and learn the features in the data better than the three neural network methods and statistical methods in the baseline methods, and make accurate predictions.

Observing Figures 6, 8, it becomes apparent that for African dataset and Hungarian dataset, the prediction results of GRGNN consistently align with the developmental trend of the original time series, albeit with varying degrees of error. This observation suggests that GRGNN, to a certain extent, can predict the developmental trends within the datasets.

The prediction errors at different step lengths are compared with the step lengths on each dataset, as shown in Figure 5 and it can be found that the prediction errors for the African data generally increase with the extension of the prediction step lengths, and the errors of the GRGNN method increase relatively less with the extension of the prediction step lengths compared with the others, which indicates that the GRGNN compared with the three neural network in the baseline methods and statistical methods to capture and learn more adequately the relationships and features among the temporal nodes of the time series. This also indicates that GRGNN learns the data in three dimensions: time domain, frequency domain and spectral domain, compared to the seven comparative forecasting methods that only learn and capture the data in the time domain, which proves that GRGNN can capture more features in the data, better grasp the overall trend of the data, and realize more accurate medium- and long-term forecasting results for the two datasets, namely, the data of the 38 countries in Africa and the data of the 20 regions in Hungary. The results demonstrate that this allows GRGNN to explore more features in the data, better grasp the general trend of the data, and thus achieve more accurate medium-term and long-term predictions for the 38 African countries’ COVID-19 dataset and the 20 Hungarian regions’ chickenpox dataset.

For the 20 Hungarian regions’ chickenpox dataset, it should be separately stated that since the data in this dataset are weekly collected, the actual predictions obtained at the same prediction step size are less than other two dataset. Therefore, as shown in Figures 5E,F, when the prediction step length is extended from 2 weeks to 3 weeks, each prediction method shows an increase in prediction error, whereas the error of each prediction method except ARIMA method shows a decreasing trend when the step length is extended from 4 weeks to 6 weeks. Meanwhile, GRGNN was able to achieve better results than the other seven comparison methods in both average RMSE and average MAE. This indicates that GRGNN and the neural network prediction methods in the baseline methods can realize the capture of the overall trend characteristics of the data, which in turn shows that the prediction accuracy will be improved when the data prediction step length is extended to a certain length, and compared with the seven comparative methods, GRGNN achieves more accurate prediction results, which indicates that GRGNN is more adequate than the other seven methods for the capture and learning of the overall trend characteristics of the data. This indicates that GRGNN is more adequate than the other seven methods for capturing and learning the general trend features of the data.

**Figure 9:**
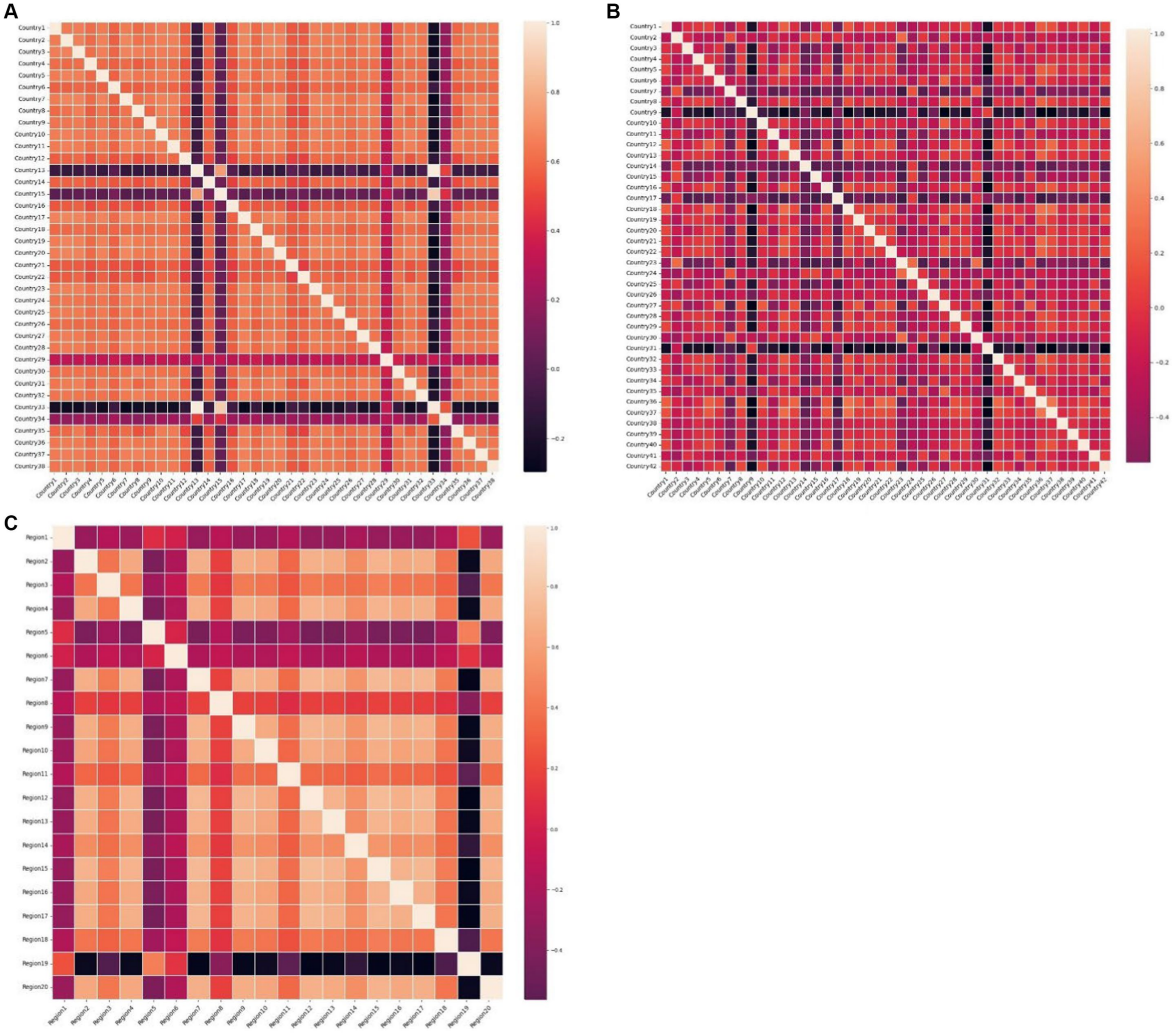
The heat maps of the weight matrices of datasets **(A)** The heat map of the weight matric of the 38 African countries’ COVID-19 dataset. **(B)** The heat map of the weight matric of the 42 European countries’ COVID-19 dataset. **(C)** The heat map of the weight matric of the 20 Hungarian regions’ Chickenpox dataset.

Finally, the GRGNN do not always make the most accurate prediction, as can be seen from Figures 5C,D, for the prediction experiments of 42 European countries, the errors of each prediction method are much larger than the errors of the prediction results for the African data, and the indicators of each prediction result under the same hyper-parameters mostly reaches 10,000 counts or even 100,000 counts, in which case the CNN-LSTM method has the best prediction results in the experiments with the prediction step lengths of 14, 21, and 28 days, and its indicators are the smallest values among the eight prediction methods, but these two metrics of CNN-LSTM become larger with the increase of the prediction step. When the prediction step is extended to 35 days, the average of CNN-LSTM is still the smallest among the eight methods, but the mean becomes sub-optimal, and the optimal value is obtained from the prediction results of GRGNN. When the prediction step size is increased to 42 days, the prediction result of GRGNN becomes optimal in both indicators. The prediction results of each prediction method in the experiment are not satisfactory in the European dataset, which may be caused by the inadequacy of the type of data collected and the insufficient amount of data collected for this phenomenon. Data inapplicability is an insurmountable problem for data-driven methods, and if the applicability of the prediction methods to the data cannot be assessed, this will greatly limit the application prospects of the prediction methods. Therefore, there is a need to discuss the applicability of GRGNN to different data:

Plotting the heatmap of the weight matrix ( W ) for each dataset in Figure 9, where the blocks in the plot represent the correlation between the time series marked by the x-axis and y-axis the lighter the color of the block is the related closer the time series are. it can be observed that the accuracy of GRGNN is linked to the correlation among time series in the datasets. In cases such as the African and Hungarian datasets in this research, where the correlation between time series is relatively close, GRGNN exhibits accurate predictions and the ability to forecast the developmental trend of the time series. However, when facing datasets like the European dataset in this research, where the correlation among time series is less pronounced, GRGNN struggles to achieve a more accurate prediction compared to other neural network methods.

We find that for the weight matrix W obtained after preprocessing of the dataset, the average of the sum of the weights of each node over the other nodes is calculated, as shown in Table 6, and it can be found that when the average value tends to 1 then the dataset yields better prediction results by GRGNN.

**Table 6:** The average node sum weights of each dataset.

	African dataset	European dataset	Hungarian dataset
average node sum weights	1.10	0.78	0.96

Therefore, we hypothesize that if the average value of the sum of the weights of each node in the weight matrix over the other nodes converges to 1, then the dataset will yield better prediction results by GRGNN. As a matter of fact, there are some researches to construct the graph by SoftMax and other methods to make the average value of the sum of the weights of each node in the weight matrix of each node to other nodes converge to 1 (40), but this hypothesis is only based on the observation of the phenomenon shown in the experimental results, and the mathematical proofs and the verification of the actual additional experiments are still need to be further supplemented.

This paper is significantly innovative: the main focus of this study is to realize the ability of the network to analyze datasets in multiple dimensions in the time, spectral, and frequency domains by introducing a GRU (Gated Recurrent Unit) layer in the GNN (Graph Neural Network) network. This gives the following advantages to the neural network used in this study: Firstly, the multiple-input multiple-output temporal prediction of multiple time series variables is more efficient compared to the single-input single-output prediction method of a single time series variable; Secondly, due to the introduction of the GRU (Gated Recurrent Unit) layer, it yields a more accurate prediction in terms of prediction accuracy; and Thirdly, as a data-driven method, it does not require human *a priori* knowledge as a basis, which makes it easy to migrate the application to the other data processing.

## 5 Conclusion

In this paper, gated recurrent units are attempted to be introduced into graph neural network, enabling graph neural networks to capture and learn features from data in three dimensions, namely, null, frequency, and time domains, which is utilized to produce notable results in the epidemic data prediction problem, which is a typical multivariate time series prediction problem. Compared with classical prediction methods, graph neural networks, as an multiple-input-multiple-output method, can quickly and easily construct graphs for multiple time series and realize effective prediction in a data-driven manner. In terms of prediction accuracy, when the predicted multivariate correlation reaches a certain level (specifically, the phenomenon observed in this study is that the closer the average of the sum of the connection weights of each node to the other nodes tends to be 1, the better the prediction results obtained from the GRGNN for the dataset), the graph neural network with the introduction of gated recurrent units can achieve more accurate predictions in medium-term or long-term forecasting.

## Data availability statement

The original contributions presented in the study are included in the article/supplementary material, further inquiries can be directed to the corresponding authors.

## Author contributions

X-dL: Writing – original draft, Writing – review & editing. B-hH: Writing – original draft, Writing – review & editing. Z-jX: Writing – original draft. NF: Writing – original draft, Writing – review & editing. X-pD: Writing – original draft, Writing – review & editing.

## Footnotes

^1^ https://www.worldometers.info/coronavirus

^2^ https://www.kaggle.com/datasets/die9origephit/chickenpox-cases-hungary

## Glossary

AMAE: Average Mean Absolute Error
ARIMA: Autoregressive Integrated Moving Average
ARMSE: Average Root Mean Square Error
AR: Autoregressive
CNN: Convolutional Neural Network
DFT: Discrete Fourier Transform
DNN: Deep Neural Network
GFT: Graph Fourier Transform
GLU: Gated Linear Unit
GNN: Graph Neural Network
GRU: Gated Recurrent Unit
HA: Historical Average
IDFT: Inverse Discrete Fourier Transform
IGFT: Inverse Discrete Fourier Transform
LSTM: Long Short-Term Memory
MAE: Mean Absolute Error
RMSE: Root Mean Square Error
RNN: Recurrent Neural Network
SMA: Simple Moving Average
VAR: Vector Autoregressive Model
WMA: Weighted Moving Average
